# Phagosomal Rupture by *Mycobacterium tuberculosis* Results in Toxicity and Host Cell Death

**DOI:** 10.1371/journal.ppat.1002507

**Published:** 2012-02-02

**Authors:** Roxane Simeone, Alexandre Bobard, Juliane Lippmann, Wilbert Bitter, Laleh Majlessi, Roland Brosch, Jost Enninga

**Affiliations:** 1 Institut Pasteur, Unit for Integrated Mycobacterial Pathogenomics, Paris, France; 2 Institut Pasteur, Research Group “Dynamics of Host-Pathogen Interactions”, Paris, France; 3 VU University, Molecular and Medical Microbiology, Amsterdam, The Netherlands; 4 Institut Pasteur, Unité de Régulation Immunitaire et Vaccinologie, Paris, France; 5 INSERM U1041, Paris, France; Weill Medical College of Cornell University, United States of America

## Abstract

Survival within macrophages is a central feature of *Mycobacterium tuberculosis* pathogenesis. Despite significant advances in identifying new immunological parameters associated with mycobacterial disease, some basic questions on the intracellular fate of the causative agent of human tuberculosis in antigen-presenting cells are still under debate. To get novel insights into this matter, we used a single-cell fluorescence resonance energy transfer (FRET)-based method to investigate the potential cytosolic access of *M. tuberculosis* and the resulting cellular consequences in an unbiased, quantitative way. Analysis of thousands of THP-1 macrophages infected with selected wild-type or mutant strains of the *M. tuberculosis* complex unambiguously showed that *M. tuberculosis* induced a change in the FRET signal after 3 to 4 days of infection, indicating phagolysosomal rupture and cytosolic access. These effects were not seen for the strains *M. tuberculosis*ΔRD1 or BCG, both lacking the ESX-1 secreted protein ESAT-6, which reportedly shows membrane-lysing properties. Complementation of these strains with the ESX-1 secretion system of *M. tuberculosis* restored the ability to cause phagolysosomal rupture. In addition, control experiments with the fish pathogen *Mycobacterium marinum* showed phagolysosomal translocation only for ESX-1 intact strains, further validating our experimental approach. Most importantly, for *M. tuberculosis* as well as for *M. marinum* we observed that phagolysosomal rupture was followed by necrotic cell death of the infected macrophages, whereas ESX-1 deletion- or truncation-mutants that remained enclosed within phagolysosomal compartments did not induce such cytotoxicity. Hence, we provide a novel mechanism how ESX-1 competent, virulent *M. tuberculosis* and *M. marinum* strains induce host cell death and thereby escape innate host defenses and favor their spread to new cells. In this respect, our results also open new research directions in relation with the extracellular localization of *M. tuberculosis* inside necrotic lesions that can now be tackled from a completely new perspective.

## Author Summary

*Mycobacterium tuberculosis* is one of the most life-threatening pathogens of all time. Despite the development of vaccines and antibiotics, this pathogen is still a major public health problem. Also the HIV epidemic has an important impact on the rise of *M. tuberculosis* infections since immunodeficient people are highly susceptible. Commonly, *M. tuberculosis* has been thought to reside in a membrane-bound compartment within its host cells during the entire infection cycle from invasion to cell death. Using a fluorescence-based method, we provide evidence that *M. tuberculosis* is able to rupture its membrane-bound compartment and gain access to the host cytosol, where it can elicit cell death. Furthermore, we show that this effect is dependent on a functional type VII secretion system named ESX-1. Most importantly, we were able to track the dynamics of infection to understand the consequences of *M. tuberculosis* phagosomal rupture. This revealed that phagosomal rupture results in cell toxicity and host cell death involving necrosis. Together, our data provide a new angle in the worldwide fight against *M. tuberculosis* and could lead to new approaches in the development of innovative treatments.

## Introduction

The intracellular localization of bacterial pathogens has important consequences for the sensing by the host and the induced host immune responses. Hence, numerous studies have investigated the intracellular niche of the microbes. Particularly, phagolysosome fusion is a central cellular mechanism used by host cells to cope with infection. Intracellular pathogens like *Mycobacterium tuberculosis* are known to avoid lysosomal fusion through the manipulation of host signal transduction pathways [1]. Following phagocytosis by a host macrophage and/or dendritic cell, *M. tuberculosis* typically resides in a phagosomal compartment that maintains many characteristics of an early endosome. The maturation towards an acidified phagolysosome is blocked or retarded by *M. tuberculosis* [2], [3]. This particularity is thought to be linked to the capacity of the bacterium to persist and replicate within macrophages [4], [5]. However, as has been recently reported by cryo-immunogold electron microscopy, other cellular mechanisms that involve translocation of *M. tuberculosis* from the phagosome into the cytosol of the infected host cell might play crucial roles at later time points of the infection process [6]. In the same study it was shown that the attenuated *Mycobacterium bovis* Bacille de Calmette et Guérin vaccine (BCG), lacking the 6kD Early Secretory Antigenic Target ESAT-6 (EsxA) and its 10kD Culture Filtrate Protein partner CFP-10 (EsxB) due to the deletion of the region of difference RD1 [7], [8] remained within the phagolysosomal compartment, similar to an *M. tuberculosis* CFP-10 transposon mutant, which was not detected within the host cytoplasm [6].

The translocation-model of van der Wel and colleagues has the potential to explain a series of observations in conflict with the often-repeated concept of *M. tuberculosis* remaining enclosed in endosomal compartments that resist maturation and acidification. Especially, it reconciled the MHC class I presentation of *M. tuberculosis* antigens, and the increased CD8 responses during *M. tuberculosis* infection [9]. Nevertheless the proposed model of phagosomal escape of *M. tuberculosis* has remained controversial due to the complexity to interpret the results entirely based on ultrastructural observations. Hence, it still awaits broad acceptance by the scientific community through independent studies and alternative experimental techniques [6], [10].

This situation prompted us to investigate the potential phagosomal escape of *M. tuberculosis* and closely related mycobacteria by the means of a recently developed fluorescence microscopy approach that proved to be a powerful tool to investigate the rupture of host endocytic vacuolar membranes during the cell invasion by Gram-negative pathogens [11], [12]. This assay requires the loading of host cells with a chemical probe that is trapped within the host cytoplasm and sensitive to fluorescence resonance energy transfer (FRET) measurements [13], [14], and a beta-lactamase activity present on the cell surface of bacteria. FRET image analysis of single infected cells can be performed in live or in fixed cells, and the intracellular localization of the bacterial pathogen can be quantified with high precision via automated image analysis tools.

We took advantage of the BlaC mediated intrinsic resistance of *M. tuberculosis*, BCG, *Mycobacterium marinum* and BlaS of *Mycobacterium smegmatis* to beta-lactam antibiotics [15]–[17] that worked in conjunction with our FRET assay. Proteome analyses have identified BlaC as a membrane-associated protein [18] containing a lipobox motif in its signal sequence that predicts membrane-anchored cell envelope localization [19], [20]. This feature allowed us to follow the cytosolic contact of selected mycobacteria over a given time course, to monitor its consequences and to link phagosomal rupture with the pathogenic potential of the tested bacterial species.

## Results

### Mycobacterial strains express and display beta-lactamase activity

The aim of our study was to analyze the capacity of selected mycobacteria to reach the cytosol during macrophage infection via a robust, sensitive and quantitative approach at the single cell level. For this, we had to adapt our FRET based reporter that was previously used to monitor the intracellular localization of *Salmonella* and *Shigella* species [11] (Figure S1) to the slowly growing mycobacteria. In the host cell, membrane permeable CCF-4-AM molecules diffuse freely across the cellular plasma membrane, are subsequently trapped in the cytosol and excluded from endosomes and other organelles by anion conversion into CCF-4 upon cytosolic esterase action. The use of this cytosolic probe relies on the bacterial expression and exposure of beta-lactamase. Cleavage of CCF-4 by beta-lactamase-expressing bacteria leads to a switch of the FRET signal from 535 nm (green) to 450 nm (blue) upon 405 nm excitation. As mycobacteria are naturally resistant to beta-lactam antibiotics, such as ampicillin, due to the constitutive expression of endogenous beta-lactamases [21], we assumed that this activity could be exploited by our assay. To ascertain that the selected mycobacterial strains showed the expected beta-lactamase activity, we first demonstrated their capacity to cleave the colorimetric beta-lactamase substrate nitrocefin (data not shown), and then evaluated their cleavage of the CCF-4 probe *in vitro* by fluorimetry. As shown in Table 1 and Figures S2A–C (in Supporting Information), all tested strains displayed the necessary enzymatic activity allowing us to follow the intracellular behavior of each strain in real-time during the process of infection of human macrophages. We decided to use the THP-1 human cell line as a host cell model because upon addition of phorbol-myristate-acetate (PMA) it differentiates from a cell with the characteristics of monocytes to one with characteristics of macrophages. Therefore, this cell line depicts a high state of differentiation. Our experimental protocol started with the infection of THP-1 cells by mycobacteria. Then, upon loading of cells with CCF-4 it was possible to discriminate exclusive phagosomal location or host cytosol access by the change of the FRET signal. Finally, using automated microscopy and dedicated image analysis algorithms, quantification was achieved on hundreds to thousands of cells for dozens of conditions in parallel.

**Table 1:** *M. marinum*, *M. smegmatis*, *M. tuberculosis* and BCG display beta-lactamase activity as shown by their ability to cleave the CCF-4 substrate *in vitro.*

	CCF-4 fluorimetric test
	450/535 nm Ratio Fold Increase
**CCF-4**	1+/−0.2
**CCF-4+BCG**	2.5+/−0.5
**CCF-4+** ***M.tuberculosis***	3.5+/−0.6
**CCF-4+** ***M.smegmatis***	11.4+/−2.1
**CCF-4+** ***M.marinum***	12.4+/−3.5
**CCF-4+BCG::pYUB412**	4.7+/−1.9
**CCF-4+BCG::RD1**	3.1+/−0.7
**CCF-4+BCG::RD1-ESAT-6Δ84-95**	2.4+/−0.2
**CCF-4** ***+M.tuberculosis***Δ**RD1**	3.5+/−1.1
**CCF-4** ***+M.tuberculosis***Δ**RD1::RD1**	2.7+/−0.6

The table summarizes the data obtained from the emission spectra measured by fluorimetry in Figure S1. The ratios reflect the intensity of the 450 nm emission peak divided by the intensity of the 535 nm emission peak upon 405 nm excitation.

Standard deviation was calculated based on 2 independent experiments.

### *M. marinum* gains rapid access to the cytosol of macrophages

**Figure 1:**
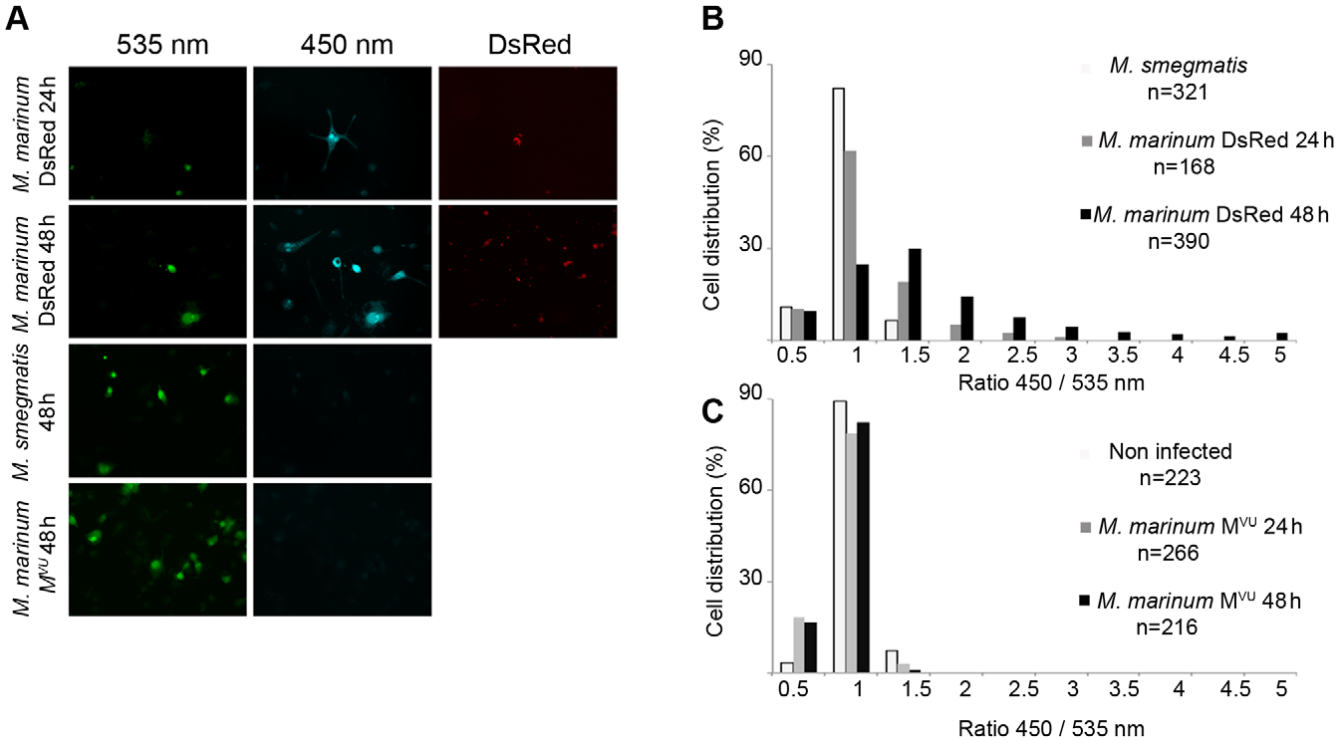
*M. marinum* is able to rupture the phagosome of THP-1 cells unlike an ESAT-6/CFP-10 secretion-deficient strain. THP-1 cells were infected with *M. marinum* expressing DsRed, *M. smegmatis* or *M. marinum* M^VU^ at a MOI of 1 for the indicated time and then loaded with CCF-4 molecule for 2 h. After PFA fixation, cells were imaged by fluorescence widefield microscope Nikon Ti with 20X objective (A). Picture acquisition was done randomly and automatically for each condition on 36 fields to follow the FRET signals and the fluorescent bacteria. Data represented in panels B and C were obtained via specialized algorithms using the Metamorph software. The plots are representative of 3 independent experiments.

At first, we compared the translocation capacities of *M. marinum* with *M. smegmatis* to evaluate the adaptability of our assay to mycobacteria. THP-1 cells were infected by *M. smegmatis* (at 37°C) or *M. marinum* expressing DsRed (at 30°C) at a multiplicity of infection (MOI) of 1 (Figure 1). As shown in Figure 1A, the *M. marinum* DsRed induced the switch of the FRET signal from 535 nm to 450 nm, indicating rupture of the phagosome and contact with the cytosol after 24 h to 48 h of infection. Strikingly, after 48 h of infection, virtually all the infected cells turned blue, and only the uninfected cells remained green. On the contrary, infection of the THP-1 cells with *M. smegmatis* did not result in a switch of the fluorescent FRET signal showing that the bacteria remained trapped within intact phagolysosomes. These results were corroborated by quantitative analysis (Figure 1B), via a dedicated algorithm segmenting individual host cells and measuring both fluorescent channels [11]. While low fluorescent ratios (around the value of 1) indicated the presence of membrane-enclosed bacteria, high ratios (above 1) showed the presence of cytosolic bacteria. Indeed, after 24 h of infection with *M. marinum* DsRed, ratios went up to 3 and even reached 5 after 48h. It is noteworthy that for *M. marinum* similar effects were already observed at rather early time points (2 h, 5 h and 20 h) (Figures S3A–F). In the case of *M. smegmatis* infection, the ratios remained low highlighting its phagolysosomal localization.

### Cytosolic contact of *M. marinum* is dependent on ESAT-6/CFP-10 secretion

We decided to take a closer look at the molecular requirements for phagolysosomal rupture of *M. marinum* by using a *M. marinum* M strain variant that was reported to be impaired for the secretion of ESAT-6 and CFP-10 and has been named *M. marinum* M^VU^ [22] (Figure S4A). As shown in Figures 1A and S3A-F, this strain did not induce a FRET shift within the infected macrophages, suggesting that it was unable to reach the cytosol of the macrophage. This effect was very strong as determined by automated image quantification of 216 randomly imaged cells after 48 h of infection (Figure 1C); not even a single infected macrophage of the inspected cells showed an increase in the cytoplasmic 450/535 nm emission ratio during the course of infection for early (Figure S2A) or late (Figure 1A) time points, although the MOIs were the same as for the ESAT-6 secretion-intact *M. marinum* M strain. In agreement with previously published data [23], [24] our results indicate that the cytosolic translocation phenotype of *M. marinum* is dependent on intact ESAT-6 secretion. This necessity is corroborated by quantitative analyses depicted in Figures 1C, S2D–F (ratio distribution was stalled at 1 throughout infection).

### Wild-type *M. tuberculosis* but not the attenuated BCG vaccine induces phagolysosomal rupture

**Figure 2:**
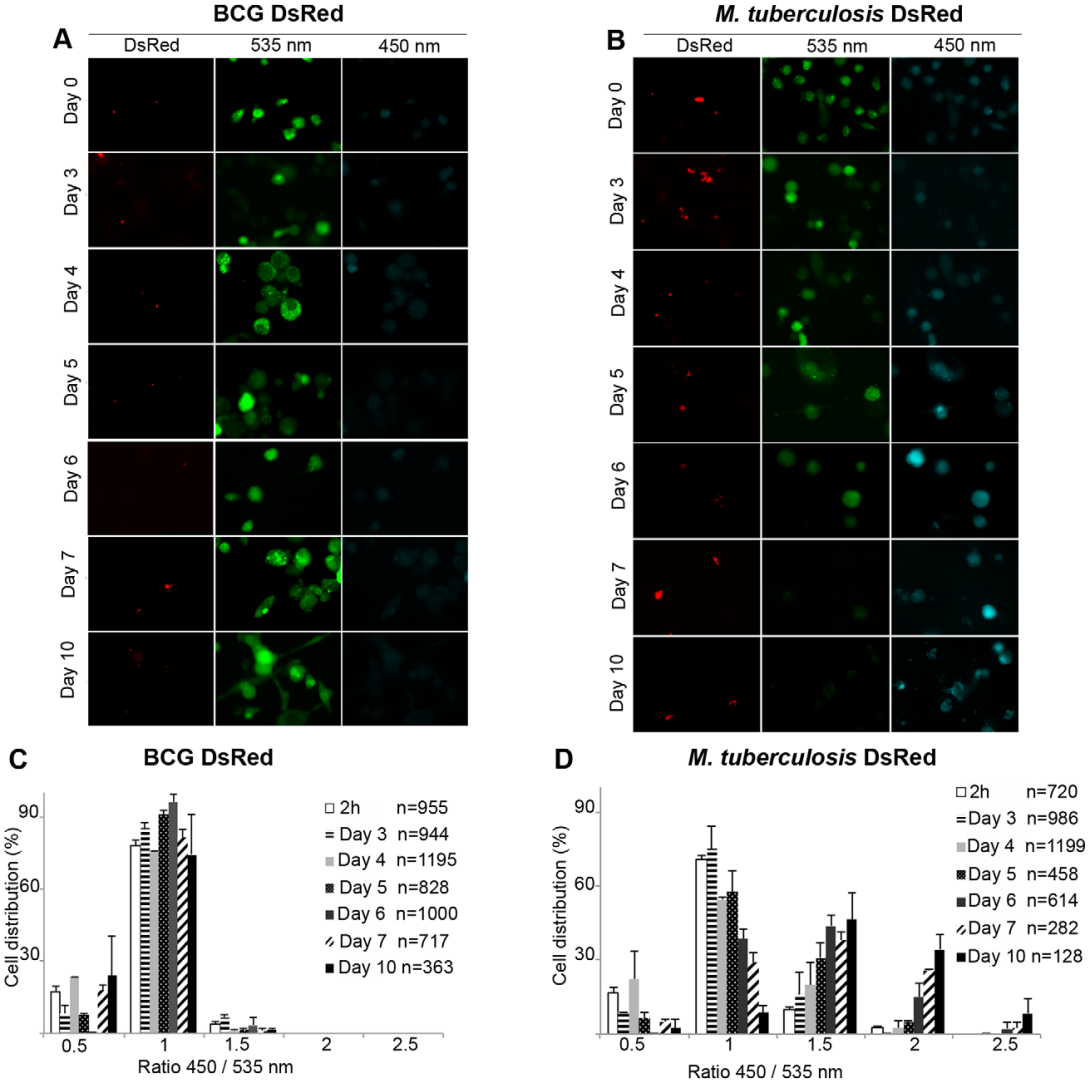
Unlike the attenuated BCG vaccine, virulent *M. tuberculosis* is able to induce phagosomal rupture in THP-1 macrophages. THP-1 cells were infected with BCG DsRed (A,C) or *M. tuberculosis* DsRed (B,D) at a MOI of 1 for the indicated time and then loaded with CCF-4 molecule for 2 h. After PFA fixation, cells were imaged using a fluorescence widefield microscope (Nikon Ti) with an 40X objective (A, B). Picture acquisition was achieved randomly and automatically for each condition on 49 fields in duplicates and further 450/535 nm intensity ratio measurements shown in panels C and D were obtained through analysis by a specialized algorithm using the Metamorph software. The plots are representative of 3 independent experiments.

We then studied the cytosolic access of the virulent *M. tuberculosis* H37Rv reference strain and the BCG vaccine strain. Unlike *M. marinum*, these tubercle bacilli display a very long infection cycle in macrophages that can last up to 1 or 2 weeks depending on the MOI. THP-1 cells were infected with BCG DsRed or *M. tuberculosis* DsRed at 37°C at an MOI of 1 for a 10 day time course. As shown in Figure 2A, BCG was unable to induce a FRET switch indicating that it remained in a membrane-enclosed compartment for the whole course of the experiment, and the same was found for the BCG::pYUB412 vector control strain (data not shown). In contrast, *M. tuberculosis* was progressively found within macrophages displaying a FRET signal switch from 535 nm to 450 nm, indicating phagosomal rupture and contact with the cytosol starting from day 3 onwards (Figure 2D). It is of note that in some cells with no apparently visible red bacteria a FRET switch was measured due to the automated focusing on the equatorial plane of the inspected cells during image acquisition. Quantification of the imaging data revealed a gradual increase of the 450/535 nm ratios over the observed time course for *M. tuberculosis* to reach 2.5 (Figure 2D), whereas ratios remained at a ratio of 1 for BCG (Figure 2C) until the end of infection at day 10. Our data obtained with BCG are very similar to the data obtained with uninfected or paraformaldhyde (PFA)-killed bacteria (Figures S5A–D). As in the experiments with *M. marinum* and *M. smegmatis* all acquired images were randomly chosen by the computer driving the microscope, and subsequently were automatically analyzed by our analytical pipeline. Taken together, our results suggest that *M. tuberculosis* is indeed able to enter into the cytosol during macrophage infection of multiple days, while BCG does not manifest this faculty, even after prolongated infection. *M. tuberculosis* thus shows a delayed but otherwise similar intracellular behavior as *M. marinum*, whose faculty to translocate into the cytosol of host cells is well established [23]–[25].

### *M. tuberculosis* ESX-1 deletion mutant resides in phagosomes

**Figure 3:**
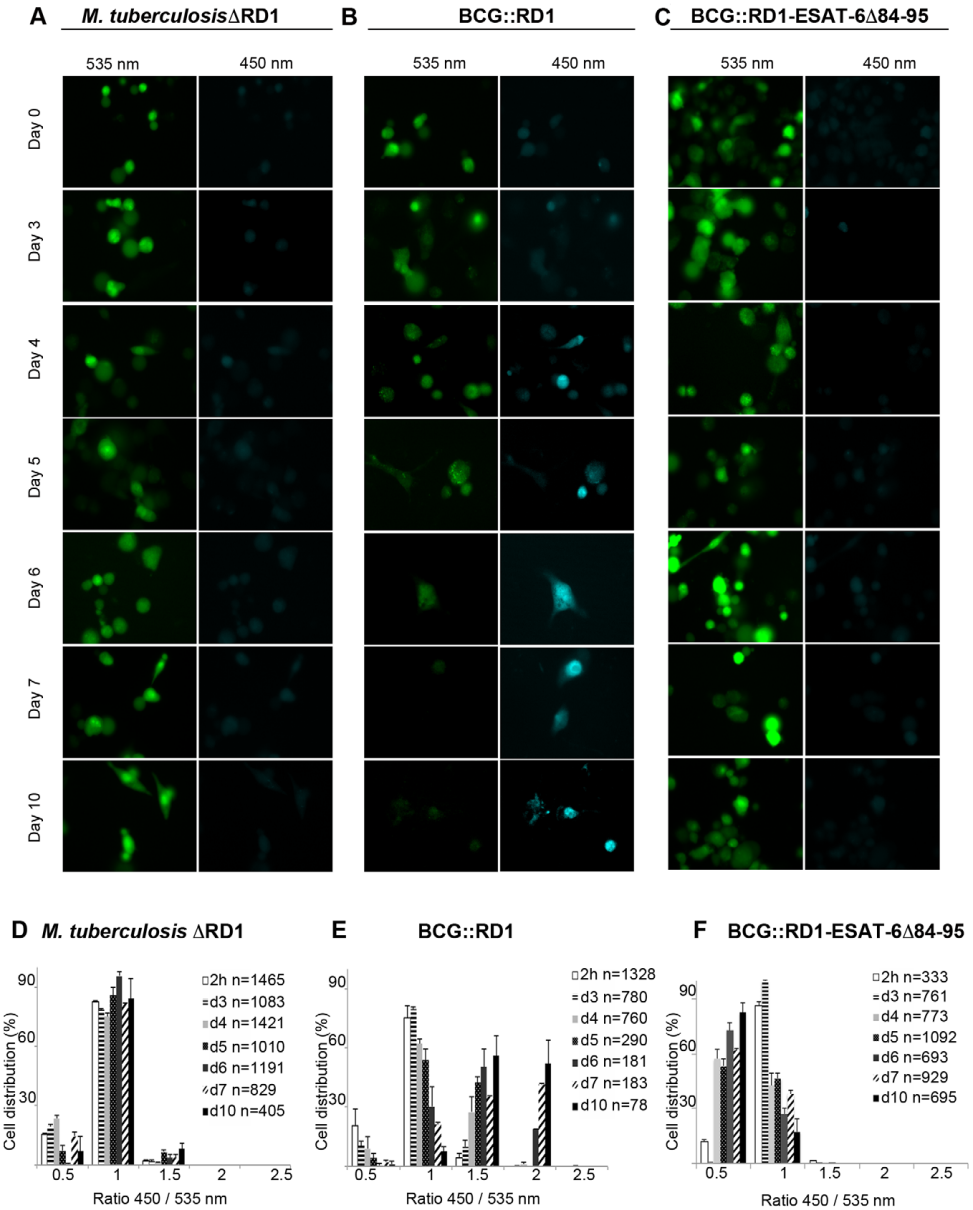
Analysis of ESX-1 deletion-, truncation- and complementation-mutants highlights the link between phagosomal rupture and functional ESX-1 secretion. THP-1 cells were infected with *M. tuberculosis*ΔRD1 (A,D), BCG::RD1 (B,E) or BCG::RD1-ESAT-6Δ84–95 (C,F) at a MOI of 1 for the indicated time and then loaded with CCF-4 molecule for 2 h. After PFA fixation, cells were imaged using a fluorescence widefield microscope (Nikon Ti) equipped with a 40X objective (A,B,C). Picture acquisition was achieved randomly and automatically for each condition on 49 fields in duplicates and further 450/535 nm intensity ratio measurement (D,E,F) was obtained through analysis by a specialized algorithm using the Metamorph software. The plots are representative of 3 independent experiments.

Based on results from previous reports and our results with the *M. marinum* ESAT-6/CFP-10 secretion null mutant (M^VU^), we decided to also test an *M. tuberculosis* ESX-1deletion mutant (*M. tuberculosis*ΔRD1) [26], which lacks a functional ESX-1 secretion system (Figure S4B). In the observed time course using an MOI of 1, we obtained similar results as for BCG, depicted by the absence of a FRET ratio switch to higher values (Figures 3A, D). This means that the ESX-1 deletion mutant bacteria reside in the phagosome until the end of our experimental time course at day 10, emphasizing that ESX-1 effectors represent secreted key factors that allow *M. tuberculosis* to gain access to the host cytosol. We also complemented *M. tuberculosis*ΔRD1 with the genomic region encompassing the ESX-1 cluster of *M. tuberculosis* (*M. tuberculosis*ΔRD1::RD1; Figure S4B) and obtained similar results as for the *M. tuberculosis* strain in the FRET analysis (data not shown).

### ESAT-6-secreting BCG::RD1 gains access to the cytosol in macrophages

In order to further investigate whether the involvement of the ESAT-6 and the ESX-1 secretion system is crucial to trigger the vacuolar rupture of mycobacteria, we tested recombinant BCG::RD1, a BCG strain that expresses and secretes ESAT-6 and CFP-10 due to the integration of a 32 kb genomic region encompassing the ESX-1 cluster of *M. tuberculosis* (Figure S4B) [27], [28]. In a time course experiment at a MOI of 1, this recombinant strain showed a similar phenotype as the one observed during *M. tuberculosis* infection. Quantitative analyses showed that a gradual increase of the number of infected cells with higher FRET ratios gradually increased starting from day 3 after infection (Figures 3B, 3E). This demonstrates that the ESX-1-complemented BCG::RD1 strain progressively gained access to the host-cell cytosol. In contrast, the use of the BCG::RD1-ESAT-6Δ84–95 strain that expresses and secretes truncated ESAT-6 (Figure S4B), showed no switch of the FRET ratio to higher values throughout the time course, indicating the absence of bacteria in the cytosol during the measured infection period (Figures 3C, 3F). These findings indicate that the cytosolic contact observed upon complementation of BCG with ESX-1 did not occur when 12 amino-acids were deleted from the secreted ESAT-6 molecule, emphasizing the importance of ESAT-6 and its C-terminal region as an effector involved in phagosomal rupture.

### Phagosomal rupture is followed by host cell death

**Figure 4:**
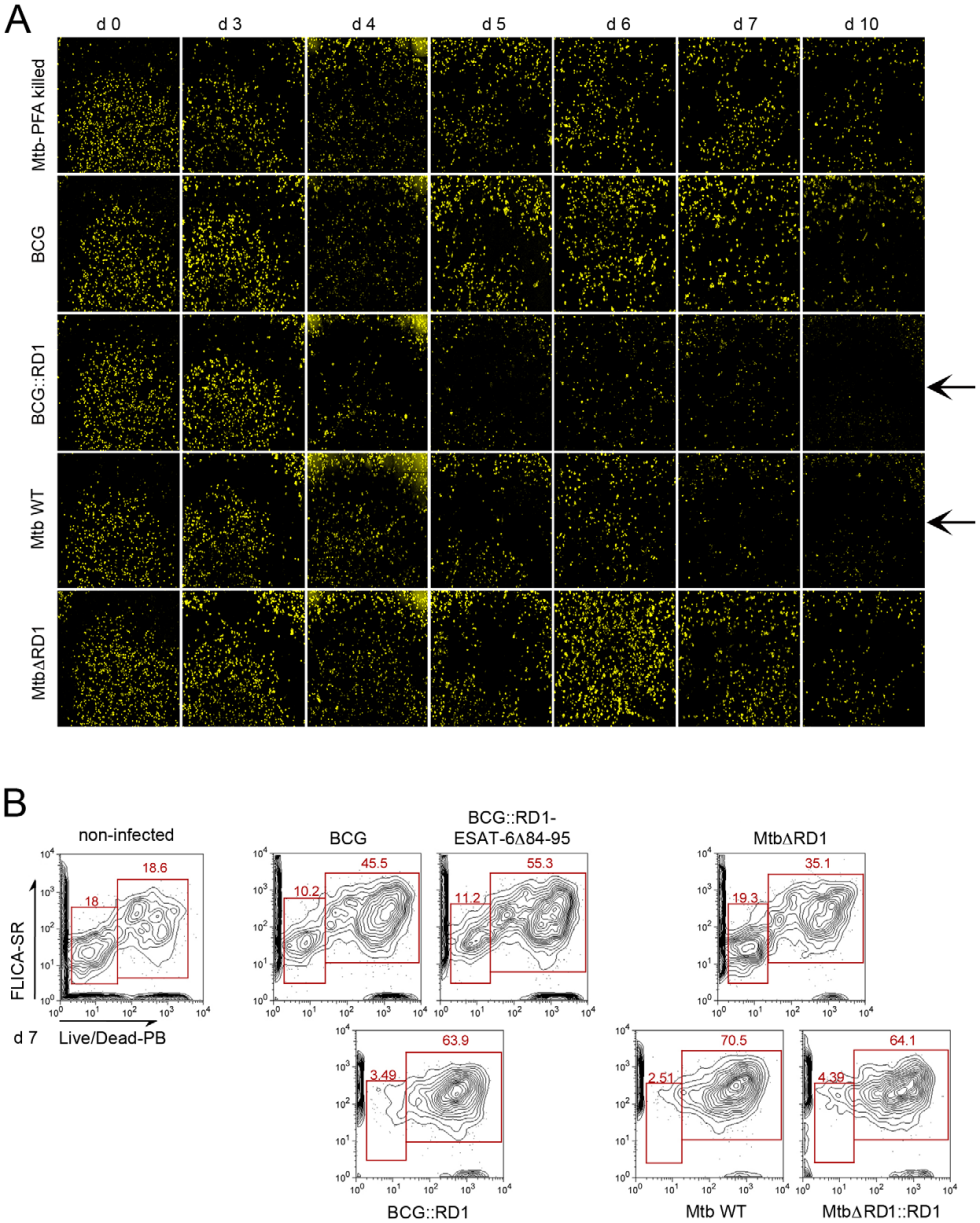
Evolution of cell numbers during the time course of infection with different *M. tuberculosis* complex members. THP-1 cells were infected with *M. tuberculosis* PFA killed, BCG, BCG::RD1, *M. tuberculosis* or *M. tuberculosis*ΔRD1 at a MOI of 1 for the indicated time. Arrows indicate the decrease of the number of cells with virulent strains. After PFA fixation, cells were stained by incubation with WGA (wheat germ agglutinin) and imaged using a Nikon Ti widefield microscope. Pictures represent a mosaic of 49 individual images from 96 well plates acquired with a 20X objective (A). Cytofluorometry analysis of THP-1 cells infected with various wild-type and recombinant *M. tuberculosis* and BCG strains at day 7 post-infection. THP-1 cells were stained with FLICA Poly-Caspases SR-VAD-FMK (apoptosis) and Live/Dead Pacific Blue (necrosis) (B).

As depicted in Figures 2D and 3E, we observed that upon infection with *M. tuberculosis* strains that cause phagosomal rupture, the total number of THP-1 cells diminished extensively over the time course of the experiment, suggesting that mycobacteria-induced phagosomal rupture and cytosolic contact leads to host cell death. Strikingly, we observed this phenomenon also for *M. marinum*, which induced cell death within 48 h (Data not shown). Furthermore, by staining THP-1 cells by the plasma membrane fluorescent marker WGA, we observed that upon infection with *M. tuberculosis* or BCG::RD1 the number of THP-1 cells decreased progressively from day 4 to day 10, which was much less pronounced for macrophages infected with mycobacterial strains that did not cause phagosomal rupture (Figure 4A). Live/Dead Pacific Blue staining in combination with FLICA poly-caspase staining and flow cytometry of THP-1 cells at day 7, when strain-specific differences were most pronounced (Figure 4A), confirmed that rupture-proficient strains caused more extensive host cell death. Only 2–4% of the cells infected with *M. tuberculosis*, *M. tuberculosis*ΔRD1::RD1 and BCG::RD1 were found viable, i.e. without any apoptosis or necrosis marker compared to 10–20% viable cells in non-infected cultures or those infected with *M. tuberculosis*ΔRD1, BCG or BCG::RD1-ESAT-6Δ84–95 (Figure 4B). Similar results were obtained when BCG and *M. tuberculosis* were tested with necrosis marker 7-AAD at day 5 by flow cytometry (Figure S6). Hence, our data support the hypothesis that cytotoxicity is linked with access to the host cytosol. Inversely, cytotoxicity was profoundly attenuated when the strains carried genetic lesions that prevented disruption of the macrophage phagolysosome.

**Figure 5:**
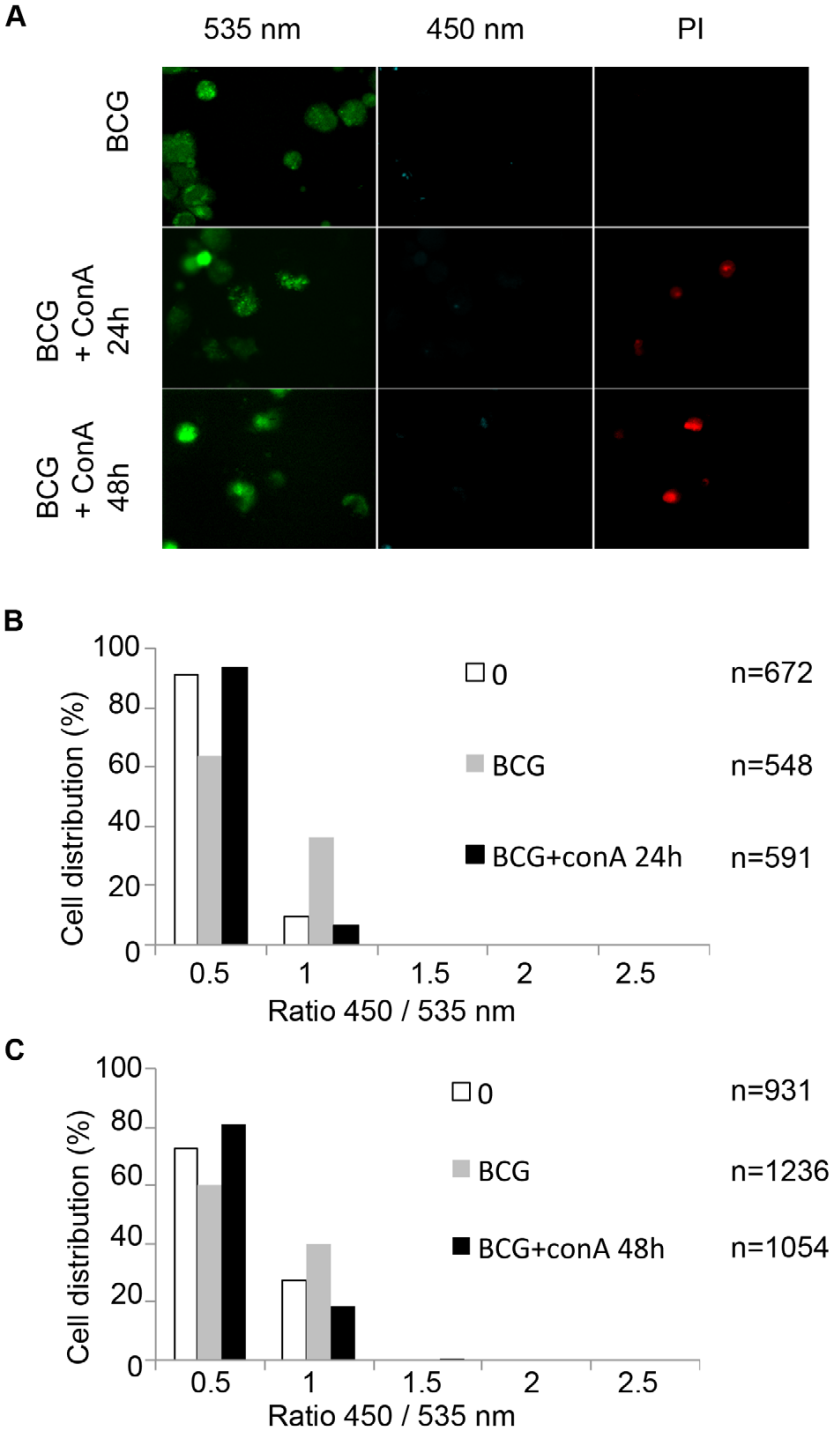
Necrosis induction does not lead to the release of mycobacteria from the phagolysosome to the cytosol. THP-1 cells were infected with BCG at a MOI of 1 for 2 days before necrosis induction using 100 µg/ml concanavalin A for 24 h or 48 h. After CCF-4 loading for 2 h, cells were incubated 5 minutes in the presence of 2 µg/ml propidium iodide (PI) and then subjected to PFA fixation. Cells were imaged using fluorescence widefield Nikon Ti microscope with 40X objective (A). Picture acquisition was achieved randomly and automatically for each condition on 49 fields and further 450/535 nm intensity ratio measurements (B,C) were obtained through analysis by a specialized algorithm using the Metamorph software. The plots were representative of 2 independent experiments.

To exclude that cell death alone leads to a FRET signal switch, we induced cell death in the THP-1 cells in multiple ways and found that no FRET change occurred under these conditions (Figures S7A, B,). We also investigated the capacity of mycobacterial strains to gain access to the cytosol upon chemical induction of host cell necrosis. For this purpose THP-1 macrophages were infected with BCG at a MOI of 1 prior to induction of necrosis by high concanavalin A concentration (100 µg/ml) for 24 h or 48 h (Figure 5). As shown in Figure 5A, concanavalin A did elicit necrosis as seen by propidium iodide nuclei staining. Nevertheless, induction of necrosis did not lead to contact of BCG with the host cytosol, as suggested by the absence of a FRET signal change during the course of the experiment (Figures 5A–C). Similarly, the concanavalin A induced necrosis conditions did not change the faculty of *M. tuberculosis* and BCG::RD1 to cause FRET switches only at later timepoints of infection. Samples taken at an early time point (24 h) exclusively showed low 450/535 nm ratios (Figures S8A–B) and the same was true for comparable infection conditions when red fluorescent *M. tuberculosis* and BCG strains were imaged (Figure S9).

Together, our data suggest that phagosomal rupture induced by mycobacterial pathogens and induction of cytotoxicity are two cellular events that are intimately linked. From the observed timescale and the performed functional assays, the sequence of events can be deduced suggesting that cytosolic contact of *M. tuberculosis* precedes and triggers host cellular death. We propose that these observations are highly relevant for a better understanding of the underlying mechanisms responsible for the pathogenicity of *M. tuberculosis* as a human pathogen.

## Discussion

Taking advantage of recently developed tools that allow the quantitative analysis of vacuolar rupture and cytosolic entry of beta-lactamase exposing bacteria, we provide unbiased evidence that *M. marinum* and *M. tuberculosis* gain access to the cytosol of infected THP-1 macrophages. These findings help to clarify the long-standing controversy of phagosomal escape of mycobacteria. While studies in the eighties and early nineties [29]–[31] showed some evidence of *M. tuberculosis* in the cytosol using traditional electron microscopy, these results remained to be confirmed by other research groups due to the lack of reliable techniques. This situation resulted in the paradigm of *M. tuberculosis* residing generally within endomembrane compartments along the endocytic route avoiding maturation and acidification of its niche. Although the model of exclusive phagosomal containment is in agreement with the fact that *M. tuberculosis* induces strong CD4^+^ T-cell responses, which is a typical feature of MHC II class presentation of peptides from phagocytosed pathogens [32], *M. tuberculosis* also elicits pronounced CD8^+^ T-cell responses [9]. The latter require proteasome-degraded cytosolic proteins to be presented via MHC I class molecules. Furthermore, *M. tuberculosis* infection also triggers type I interferon responses [33], and NLRP3 inflammasome activation, where ESAT-6 has been proposed to facilitate the diffusion of immunomodulatory PAMPS (Pathogens-Associated Molecular Patterns) to the cytosol resulting in enhanced caspase 1 activation [34]. Thus, immunological data clearly suggest that mycobacterial constituents appear and are processed within the cell host cytoplasm during infection.

A plausible way to interpret these findings takes into account that intracellular mycobacterial pathogens rupture the phagosomal membrane and translocate to the cytosol at some stages of the infection and thereby initiate the cytosolic recognition pathways. Evidence for such mycobacterial translocation was reported for *M. marinum*, which behaved similar to *Shigella flexneri* forming actin comet tails inside the host cytoplasm [25]. More recently, potential phagosomal escape of *M. marinum* was also linked to the formation of the ejectosome [35], and/or the trapping of the pathogens within septin cages [36].

For *M. tuberculosis* novel ways of sample preparation under cryo-conditions and immuno-gold staining were employed to investigate the intracellular pathogen localization challenging the common view of the exclusive phagosomal localization of *M. tuberculosis* [6]. However, the subject has remained controversial, and the study of phagosomal rupture using independent techniques is of major scientific interest [10]. In this respect, our fluorescence microscopy-based approach for live and fixed samples bring new quantitative insight into this phenomenon using an alternative simple, specific and sensitive experimental approach. Our data provide evidence that phagosomal rupture and contact with the host cytoplasm occurs at a species time course during the infection process.

This observation is even more significant as such rupture was never observed for the different mutant strains impaired for ESAT-6 and CFP-10 secretion (Figures 2A, 3A), thereby demonstrating that the observed effects did not occur at random, but in a biologically relevant fashion. Strikingly, BCG devoid of the ESX-1 secretion system due to deletion of the RD1 region [7] was unable to induce a change in the 450/535 nm ratio, whereas the ESX-1 complemented BCG::RD1 strain under the same experimental conditions clearly induced a change in the ratio from green to blue, indicating cytosolic contact (Figures 3B, 3E). These results extend previous reports on the potential involvement of the ESX-1 system showing that the ESX-1 system is a central component required for phagosomal rupture. Belonging to the recently described type VII secretion systems [37], [38], ESX-1 is one of the five ESX systems in *M. tuberculosis*, designated ESX-1 to ESX-5, that is well known for its involvement in virulence and specific immune responses [39]. Indeed, *M. tuberculosis, M. bovis* and/or *M. marinum* mutants lacking an intact ESX-1 secretion system or its substrates have been shown to be attenuated in cultured macrophages and animal models of infection, thereby exhibiting defects in cell to cell spread, altered cytokine profiles [23], [26], [40]–[42] or phagosome maturation arrest [43]. Furthermore, it has been suggested that ESAT-6 secreted by *M. marinum* could play a direct role in producing pores in membranes of the vacuoles containing mycobacteria, facilitating the escape of *M. marinum* from the vacuole to the cytosol and cell to cell spread [44]. Similar observations were made with purified ESAT-6 from *M. tuberculosis* that destabilized and lysed artificial lipid bilayers and liposomes [26], [45].

A more distantly related ESX-1 system is also present in *M. smegmatis,* where it is thought to serve as a putative conjugation system [46]. As *M. smegmatis* is an avirulent, saprophytic and fast-growing mycobacterium that did not show any phagosomal translocation in our assay, it is possible that ESAT-6 and CFP-10 from *M. smegmatis*, which share only 60–70% amino acid identities with their homologues from *M. tuberculosis* might not fulfill the same function(s) and/or might miss potential interaction partners. Inspection of available genome data showed that *M. smegmatis* is not the only non-pathogenic fast-growing mycobacterium carrying an orthologous ESX-1 system. Other species, such as *Mycobacterium vanbaalenii* (GenBank: CP000511), *Mycobacterium gilvum* (CP000656), *Mycobacterium sp*. JLS (GenBank: CP000580), *Mycobacterium sp*. KMS (GenBank: CP000518) and *Mycobacterium sp*. MCS (GenBank: CP000384) also encode ESX-1 systems that resemble ESX-1 in *M. tuberculosis* in gene content and gene order. In contrast, these fast-growing mycobacterial species lack orthologs of the EspACD locus that in *M. tuberculosis* is involved in the regulation of ESX-1 mediated virulence [47]–[50]. As the EspACD locus is present in *M. marinum*, *M. leprae*, and *M. tuberculosis*, which have all been described as translocation-proficient mycobacteria [6], [24], [25], it might well be that phagosomal rupture requires interaction of ESX-1 and EspACD proteins. The CCF-4 based experimental approach presented here will be helpful to investigate this question in the near future.

We show that mycobacterial phagosomal rupture precedes host cell death (Figures 4, 5, S8 and S9). Using necrosis markers, like propidium iodide we found that infected host cells harboring cytoplasmic bacteria tested positive for necrosis within one to two days upon FRET signal switch, however cells appeared as “empty bags” at the very late time points complicating the interpretation of the results. Flow cytometry confirmed that most of *M. tuberculosis* and BCG::RD1 infected cells stained positively for necrosis and/or apoptosis markers, whereas this effect was substantially reduced for infection with *M. tuberculosis*ΔRD1 and BCG (Figures 4B, S6). Triggering cell death in infected macrophages is in agreement with studies suggesting the role of the ESX-1 system during this event [24], [26], [51]. Independent studies have investigated the different mechanisms of host cell death that may have an impact on triggering the immune response [52]. Depending on the individual cell cycle of each of the involved bacteria, it might be sufficient if only a few bacteria become cytosolic to induce the initiation of cell death supporting the hypothesis of Fortune and Rubin on phagosomal escape of *M. tuberculosis*, who suggested that the cytosol might only be a brief stop on the way to escape from the intracellular environment altogether [10]. Importantly, in many hosts, large numbers of bacteria have been found to be extracellular, such as those found in necrotic caseous lesions. Thus, the paradigm of intracellular growth might represent only a part of the life cycle of the infectious organisms [10]. Such a scenario would also explain why only a certain percentage of bacteria was previously found in the cytosol by EM and other techniques due to the experimental procedures.

In conclusion, our presented data on the ESX-1-dependent potential of *M. marinum,* BCG::RD1 and *M. tuberculosis* to change the FRET signal in an ESX-1-dependent manner is in excellent agreement with previous studies that have suggested an involvement of ESAT-6 in membrane and/or cell lysis. The similarities with the FRET changes caused by IpaB-secreting *Shigella flexneri* [11], [53] suggest that these mycobacterial strains also gain cytosolic access via rupture and disassembly of phagosomal membranes, but at later stages of infection. However, taking into consideration that the reported ESX-1-dependent type I interferon response to infection with *M. tuberculosis* occurs already 24 h post infection [33], it is plausible that rupture of the phagosomal membrane is preceded and/or initiated by pore-forming activity of ESAT-6, which might allow small signalling molecules such as cyclic diadenosine monophosphate (c-di-AMP) [54] or other PAMPs to translocate prior to the escape of entire bacteria and thereby trigger an early cytosolic host response. In any case, the ESX-1 mediated access to the cytosol seems to represent a major switch for many cellular parameters and the resulting immune responses [39], [55], which are thus substantially different between BCG and *M. tuberculosis*. Hence, infection experiments using BCG as a model organism might have the disadvantage of potentially missing out on this part of cellular responses that are linked to the cytosolic access of the bacteria. The BCG::RD1 strain seems to represent a good compromise, as it is much less virulent than *M. tuberculosis*, but due to the access to the cytosol still elicits ESAT-6 specific immune responses similar in quality and quantity to *M. tuberculosis* [9]. This finding seems also to be an important feature for the enhanced protective efficacy of BCG::RD1 or other ESX-1 complemented vaccine strains relative to BCG alone [27], [56]. In a more practical sense, our findings have direct relevance for the design of new vaccines and the potential development of new therapeutic intervention strategies that might target the ESX-1 secretion system. It is clear from our study that a more effective vaccine strain should have the capacity to access to the cytosol of target cells in order to induce the series of immunologic responses ordinary BCG is unable to induce. A potential combination of the properties of BCG and *M. marinum* might represent one novel route to explore. Finally, the presented CCF-4 assay might also represent a powerful new system that can be automated for cell-based screening of larger compound libraries [57] in order to identify molecules that can block phagosomal rupture and thereby a whole series of events that are linked to the pathogenicity of one of the most deadly pathogens of mankind.

## Materials and Methods

### Bacterial strains, growth conditions

Wildtype and mutant strains of *M. tuberculosis* (H37Rv), *M. bovis* BCG Pasteur 1173P2, *M. marinum* M^VU^, *M. marinum* M Dsred and *M. smegmatis* (mc^2^ 155) were grown in 7H9 liquid medium (Difco) supplemented with albumin-dextrose-catalase (ADC, Difco) or on solid Middlebrook 7H11 medium (Difco) supplemented with oleic acid-albumin-dextrose-catalase (OADC). With the exception of *M. marinum*, which was grown at 30°C, all other strains were cultivated at 37°C. *M. tuberculosis* or *M. marinum* heat killed strains were obtained by heating the sample at 95°C for 30 min. PFA-fixed *M. tuberculosis* or *M. marinum* bacteria were obtained after incubation with PFA 4% for 30 min. If required, the media were supplemented with 20 µg/ml of kanamycin or 50 µg/ml of hygromycin. *Shigella flexneri* (M90T-AfaI) was grown in BTCS and *Salmonella typhimurium* was grown in LB broth.

### Mycobacterial infections

The human pro-monocytic cell line THP-1 was maintained in RPMI 1640 glutamax and 10% heat-inactivated fetal bovine serum (FBS) at 37°C under an atmosphere containing 5% CO_2_. For differentiation into macrophages, cells were plated into 96-well Greiner plates that work for fluorescence microscopy (3x10^4^ cells/well) and treated with 20 ng/ml of PMA (Sigma) for 72 h or were seeded into 24-well plates for flow cytometry (5x10^5^ cells/well). Before use, cells were washed twice with fresh medium. *M. tuberculosis* and BCG strains were grown to mid-log phase in 7H9 containing albumin dextrose catalase (ADC, Difco) medium at 37°C. Cultures were harvested, washed, resuspended in PBS and gently sonicated to avoid clumping. The concentration of each strain was determined by OD600 measurement. Then PMA-differentiated THP-1 cells were infected with *M. tuberculosis* and BCG suspensions at a MOI of 1∶1 in RPMI medium during for 2 h at 37°C with 5% CO_2_. After 2 h, the medium was removed and cells were washed 3 times to remove extracellular bacteria before the addition of fresh medium. Time course measurements evaluating phagosomal rupture were done at days 3, 4, 5, 6, 7 and 10.

*M. marinum* strains were grown to mid-log phase in 7H9 containing ADC at 30°C. Cultures were harvested, washed, resuspended in PBS and then filtered though a syringe. The concentration of each strain was determined by OD600 measurement. Then PMA-differentiated THP-1 cells were infected with *M. marinum* suspensions at an MOI of 1∶1 in EM medium (120 mM NaCl, 7 mM KCl, 1.8 mM CaCl_2_, 0.8 mM MgCl_2_, 5 mM glucose and 25 mM Hepes at pH 7.3) for 2 h at 30°C. After 2 h, the medium was removed and cells were washed 3 times to remove extracellular bacteria before the addition of fresh EM medium containing Hepes and 10% FBS. Time course measurements to monitor the *M. mariunum* phagosomal rupture were done at day 0, 1 and 2 to monitor the *M. mariunum* phagosomal rupture.

### *Shigella flexneri* and *Salmonella typhimurium* infection

Ampicilline-resistant bacteria were incubated overnight at 37°C with shaking, diluted 1/100 in BTCS (for *Shigella M90T-AfaI*) or LB broth (for *Salmonella typhimurium*), and incubated in the same conditions for 2,25 h. The cultures were washed with PBS. *Salmonella typhimurium* and *Shigella flexneri* were incubated with 0.1 mg/ml beta-lactamase (Sigma) or 0.1 mg/ml Atto594 beta-lactamase, respectively, for 10 min. After PBS washing, cultures were diluted in 10% FBS-containing DMEM/F12 (Gibco) and added to the cells at a MOI of 100 at 37°C.

### Labelling of soluble beta-lactamase

Labelling of beta-lactamase (Sigma) was obtained using Atto594 protein labelling kit (Fluka).

### CCF-4 assay to monitor mycobacterial vacuolar rupture

At the successive stages of the time course measurements, a mix containing 50 µM CCF-4 substrate (Invitrogen) in EM containing 2.5 µM probenicid was added for 2 h at RT in the dark. Cells were washed with PBS containing 2.5 µM probenicid before fixing with PFA 4% for 30 min at RT in the dark. Cells were washed before performing directly fluorescence imaging or additional cellular staining.

### Fluorescent tools and fluorescence imaging

The necrotic marker propidium iodide (Fluka) was used just before fixation for 5 minutes at 2 µg/ml in PBS in the dark. The cell membrane marker WGA 647 (Invitrogen) was used after fixation at 2 mg/ml in PBS in the dark for 30 min. Samples were imaged using an automated inverted fluorescent widefield microscope Nikon Ti with 20X or 40X objective driven by Metamorph. Picture acquisition was achieved randomly and automatically for all measurements of this study on 36 to 49 fields per condition. Samples were excited at 405 nm, and emission was followed to determine the 450/535 nm intensity ratio. Automated image analysis including cell segmentation and quantification was achieved using dedicated journals written for Metamorph [11]. The used Metamorph journals are available upon request.

### Flow cytometry analysis

Two different flow cytometry assays were performed to evaluate cell death caused by different *M. tuberculosis* and BCG strains at later timepoints of infection. THP-1 cells were labeled with FLICA Poly-Caspases (SR-VAD-FMK) marker (Immunochemistry Technologies), which detects apoptotic cells, and LIVE/DEAD Pacific Blue Fixable Dead Cell Stain Kit (Invitrogen), which detects necrotic cells, according to the manufacturer recommendations. The stained cells were fixed overnight with 4% PFA and cytometric data were acquired on a Cyan system, using the Summit software (Beckman Coulter, Villepinte, France). Data were analyzed with FlowJo software (Tree Star, OR, USA). A parallel experiment was performed for BCG and *M. tuberculosis* strains using 7-AAD staining (BD-Pharmingen) according to the manufacturer's recommendations. The stained cells were fixed overnight with 4% PFA and analyzed using a FACS ARIA III flow cytometer and FlowJo software (Tree Star, OR, USA).

### Biochemical analysis

*M. tuberculosis* strains or *M. bovis* BCG strains were grown in liquid medium, at pH 7 for 7 days and 4 days for *M. marinum* strains. Cultures were harvested by centrifugation. The supernatant was recovered after filtration through 0.22 µm pore size filters (Millipore) and protease inhibitors were added (Complete EDTA Free; Roche Diagnostics GmbH, Mannheim, Germany). The cell pellet was washed twice and resuspended in PBS. Cells were ruptured by shaking with 106-µm acid washed-glass beads (Sigma) for 8 min at speed 30 in a Mill Mixer (MM300; Retsch GmbH, Haan, Germany). The whole cell lysate consisting of the supernatant fraction recovered after filtration and debris were removed by centrifugation at 17000 g for 30 min. Total protein concentrations were determined by using Nanodrop. Samples were subjected to NuPAGE Novex Bis-Tris pre-cast gel (12%) (Invitrogen) before blotting on iBlot Gel Transfer Stacks Mini Nitrocellulose (Invitrogen) with iBlot Dry Blotting System. Immunoblotting was performed with mouse monoclonal anti-ESAT6 antibody (Hyb 76-8, Antibodyshop) and mouse monoclonal anti-GroEL2 antibody as lysis control as described [58]. Antibodies against GroEL2 were received as part of National Institutes of Health, National Institute of Allergy and Infectious Diseases contract entitled “Tuberculosis Vaccine Testing and Research Materials,” awarded to Colorado State University.

### Determination of beta-lactamase activity *in vitro*

Beta-lactamase activity was assayed spectrophotometrically with 100 mM nitrocefin (Calbiochem) in 0.1 M sodium phosphate buffer at 37°C. Hydrolysis was monitored at 486 nm using a UV spectrophotometer. The molecular extinction coefficient of hydrolyzed nitrocefin at 486 nm is 20500 M^−1^cm^−1^. The rate of nitrocefin hydrolysis by each protein was expressed as micrograms of nitrocefin hydrolysed per minute per microgram of protein.

Fluorimetric assays were performed in 1 ml PBS containing 50 µg/ml porcine esterase liver extracts (Sigma) and 100 nM CCF-4-AM liveblazer (Invitrogen). The esterase was added to cleave off the –AM ester moieties yielding fluorescent CCF-4. Bacteria were washed with PBS and added to the mixture for 12 h at 37°C in the dark. Soluble lactamase 1 mg/ml was used as a positive control. Fluorescence intensity measurements were then performed using PTI Quantamaster fluorimeter in 1ml quartz cuvettes.

### Induction of cell death

Staurosporine (Sigma), cycloheximide (Sigma) and TNF-α (RD systems) were used for inducing apoptosis whereas concanavalin A (Sigma) and H_2_O_2_ (Sigma) were used for inducing necrosis.

## Supporting Information

Figure S1 **Time course of CCF-4 FRET signals in THP-1 macrophages treated with soluble Atto594-lactamase, infected by M90T-AfaI** ***Shigella flexneri*** **or DsRed-** ***Salmonella typhimurium.*** THP-1 cells were incubated with Atto594-lactamase 100 µg/ml (A,B), infected by Atto594-lactamase loaded *Shigella flexneri* (C,D) or infected with DsRed-*Salmonella typhimurium* (E,F) for the indicated time and then loaded with the CCF-4 molecule for 2 h. After PFA fixation, cells were imaged on a fluorescence widefield microscope (Nikon Ti) with a 40X objective (A,C,E). Picture acquisition was achieved randomly and automatically for each condition on 49 fields in duplicates and further 450/535 nm intensity ratio measurements (B,D,F) were obtained through analysis by specialized algorithms on Metamorph software. The plots were representative of 3 independent experiments.
(TIF)

Figure S2 **Different mycobacteria strains are able to cleave the CCF-4 probe** ***in vitro.*** *M. marinum, M. smegmatis* (A), BCG, *M. tuberculosis* (B), BCG::RD1, BCG::RD1-ESAT-6Δ84-95, BCG::pYUB412, *M. tuberculosis*ΔRD1 and *M. tuberculosis* ΔRD1::RD1 (C) were put in contact with CCF-4 for 12 h at 37°C. Then, emission spectra from 425 to 550 nm were obtained upon 405 nm excitation. Soluble lactamase 1 mg/ml was used as a positive control (A). Standard deviation was calculated based on 2 independent experiments
(TIF)

Figure S3 **Cytosolic access of** ***M. marinum*** **depends on secretion of ESAT-6/CFP-10 effectors at early time points of infection.** THP-1 cells were infected with *M. marinum* heat killed, *M. marinum* or *M. marinum* M^VU^ at a MOI of 1 for 2 h (A,D), 5 h (B,E) or 20 h (C,F). Cells were then loaded with the CCF-4 molecule for 2 h. After PFA fixation, cells were imaged on a fluorescence widefield microscope (Nikon Ti) with a 40X objective (A,B,C). Picture acquisition was achieved randomly and automatically for each condition on 36 fields and further 450/535 nm intensity ratio measurements (D,E,F) were obtained through analysis by specialized algorithm on Metamorph software. Experiments were repeated 3 times with similar results.
(TIF)

Figure S4 ***In vitro*** **expression and secretion of ESAT-6 in different mycobacterial strains.** Cell pellets and supernatants were subjected to SDS-PAGE and tested by Western blotting using a monoclonal anti-ESAT6 antibody or an anti-Groel2 antibody as lysis control. *M. marinum* M^VU^, *M. marinum* M DsRed; *M. tuberculosis* H37Rv (A), BCG::pYUB, BCG::RD1, BCG::RD1-ESAT-6Δ84-95, *M. tuberculosis* H37Rv, *M. tuberculosis*ΔRD1, *M. tuberculosis*ΔRD1::RD1 (B).
(TIF)

Figure S5 **Time course of CCF-4 FRET signals in uninfected and PFA killed** ***M. tuberculosis*** **treated THP-1 cells.** THP-1 cells were infected with *M. tuberculosis* DsRed PFA killed (A,C) or not infected (B,D) at a MOI of 1 for the indicated time and then loaded with the CCF-4 molecule for 2 h. After PFA fixation, cells were imaged on a fluorescence widefield microscope (Nikon Ti) with a 40X objective (A,B). Picture acquisition was achieved randomly and automatically for each condition on 49 fields and further 450/535 nm intensity ratio measurements (C,D) were obtained through analysis by specialized algorithms on Metamorph software. The plots were representative of 3 independent experiments.
(TIF)

Figure S6 **Evaluation of cell death in THP-1 cells by cytofluorometry.** Cytofluorometry analysis of THP-1 cells infected with wild-type *M. tuberculosis* and BCG strains at day 5 post-infection. THP-1 cells were stained with 7-AAD (BD Pharmingen) (necrosis).
(TIF)

Figure S7 **Apoptosis and necrosis do not influence the cleavage of the CCF-4 probe in THP-1 macrophages.** THP-1 cells were subjected to different treatments for 18 h in order to induce either apoptosis or necrosis. Apoptosis was induced using 4 µM staurosporine, 5 µg/ml cycloheximide or 200 ng/ml TNF-α. Necrosis was induced using 0.1% H_2_O_2_, 100 µg/ml concanavalin A or cycloheximide/TNF-α combination. Cells were then loaded with the CCF-4 probe for 2 h. After PFA fixation, cells were imaged on a fluorescence widefield microscope (Nikon Ti) with a 40X objective (A). Picture acquisition was achieved randomly and automatically for each condition on 49 fields and further 450/535 nm intensity ratio measurements (B) were obtained through analysis by specialized algorithms on Metamorph software
(TIF)

Figure S8 **Necrosis induction does not influence the subcellular localization of mycobacteria.** THP-1 cells were non infected (control) or infected with BCG, BCG::RD1, *M. tuberculosis* or *M. tuberculosis*ΔRD1 at a MOI of 1 for 2 h. Then, necrosis was induced using 100 µg/ml concanavalin A for 24 h. Cells were then loaded with CCF-4 molecule for 2 h. After PFA fixation, cells are imaged by fluorescent widefield microscope Nikon Ti with 40X objective (A). Picture acquisition was achieved randomly for each condition on 49 fields and further 450/535 nm intensity ratio measurement (B) was obtained through analysis by a specialized algorithm on Metamorph software.
(TIF)

Figure S9 **Necrosis induction in BCG or** ***M. tuberculosis*** **infected cells does not result in bacterial contact with host cytoplasm.** THP-1 cells were non infected (control) or infected with BCG DsRed or *M. tuberculosis* DsRed at a MOI of 1 for 2 h. Then, necrosis was induced using 100 µg/ml concanavalin A for 24 h. Cells were then loaded with CCF-4 molecule for 2 h. After PFA fixation, cells were imaged by fluorescence widefield microscope Nikon Ti with 40X objective.
(TIF)
