# Supplementary figures and images for: Phagosomal Rupture by Mycobacterium tuberculosis Results in Toxicity and Host Cell Death
Source: PLoS Pathog. 2012 Feb 2;8(2):e1002507. doi: 10.1371/journal.ppat.1002507 (PMC3271072; doi:10.1371/journal.ppat.1002507)

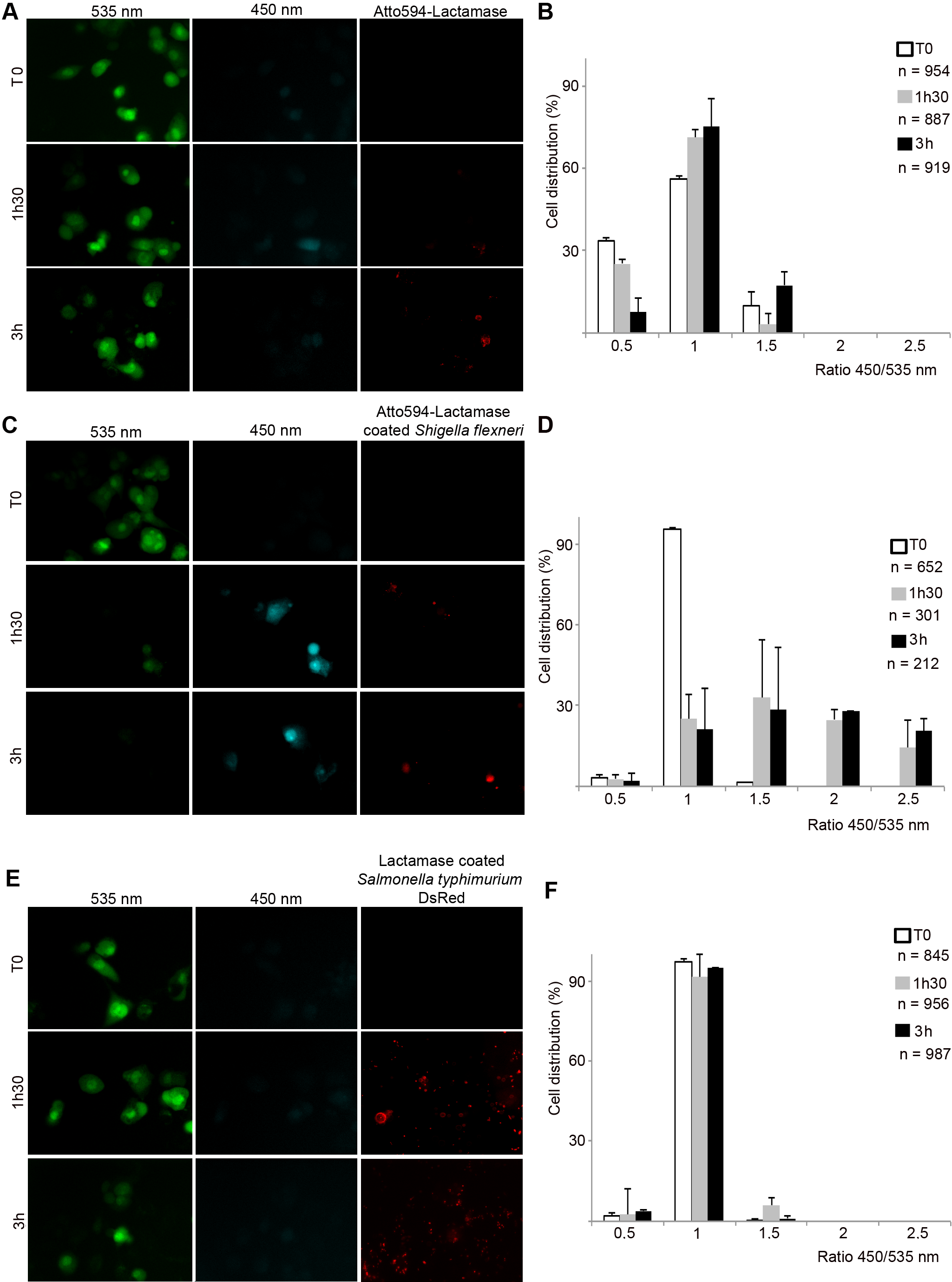

Supplement: Figure S1 — Time course of CCF-4 FRET signals in THP-1 macrophages treated with soluble Atto594-lactamase, infected by M90T-AfaI Shigella flexneri or DsRed- Salmonella typhimurium. THP-1 cells were incubated with Atto594-lactamase 100 µg/ml (A,B), infected by Atto594-lactamase loaded Shigella flexneri (C,D) or infected with DsRed-Salmonella typhimurium (E,F) for the indicated time and then loaded with the CCF-4 molecule for 2 h. After PFA fixation, cells were imaged on a fluorescence widefield microscope (Nikon Ti) with a 40X objective (A,C,E). Picture acquisition was achieved randomly and automatically for each condition on 49 fields in duplicates and further 450/535 nm intensity ratio measurements (B,D,F) were obtained through analysis by specialized algorithms on Metamorph software. The plots were representative of 3 independent experiments. (TIF) [file ppat.1002507.s001.tif]

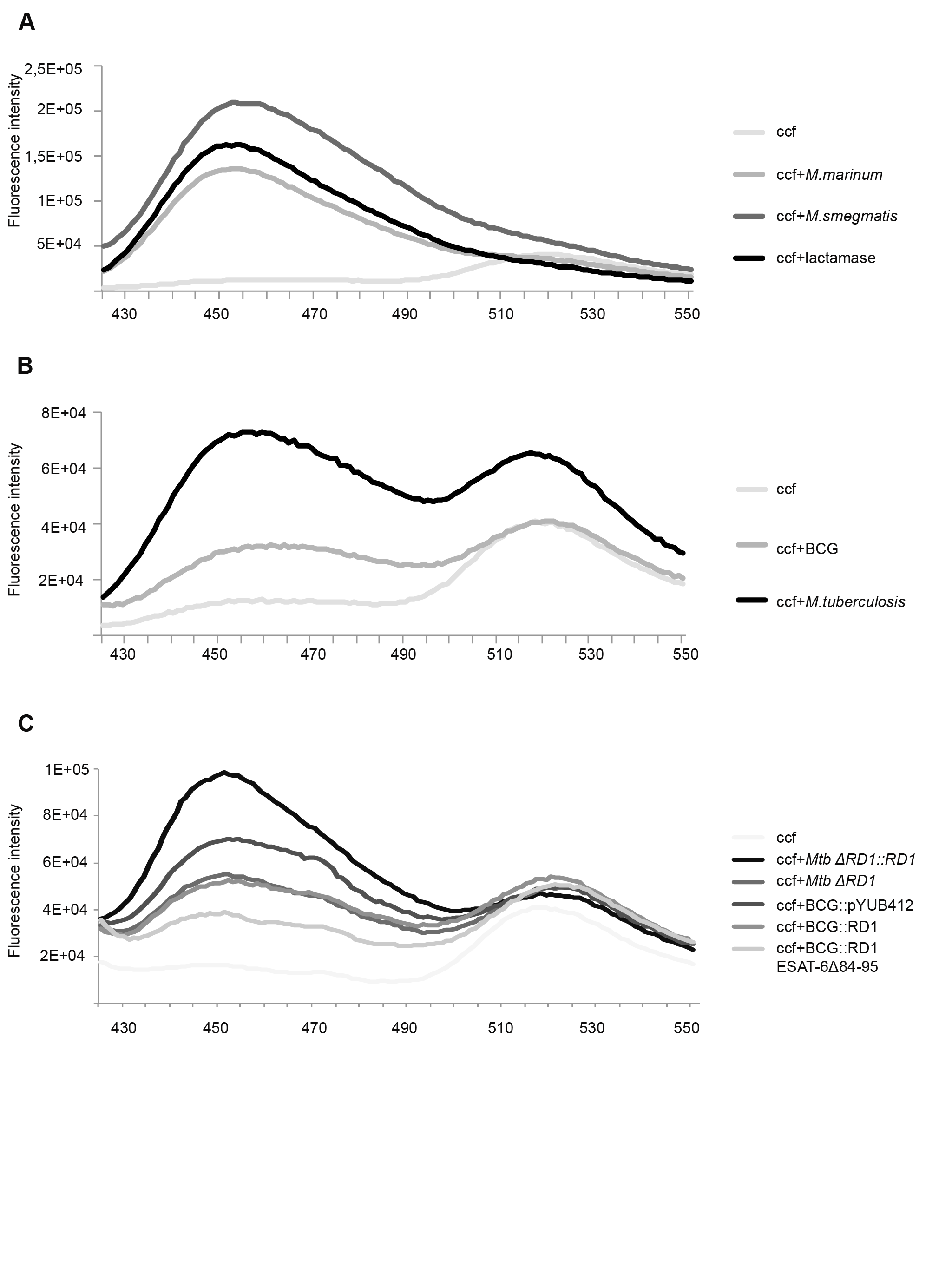

Supplement: Figure S2 — Different mycobacteria strains are able to cleave the CCF-4 probe in vitro. M. marinum, M. smegmatis (A), BCG, M. tuberculosis (B), BCG::RD1, BCG::RD1-ESAT-6Δ84-95, BCG::pYUB412, M. tuberculosisΔRD1 and M. tuberculosis ΔRD1::RD1 (C) were put in contact with CCF-4 for 12 h at 37°C. Then, emission spectra from 425 to 550 nm were obtained upon 405 nm excitation. Soluble lactamase 1 mg/ml was used as a positive control (A). Standard deviation was calculated based on 2 independent experiments (TIF) [file ppat.1002507.s002.tif]

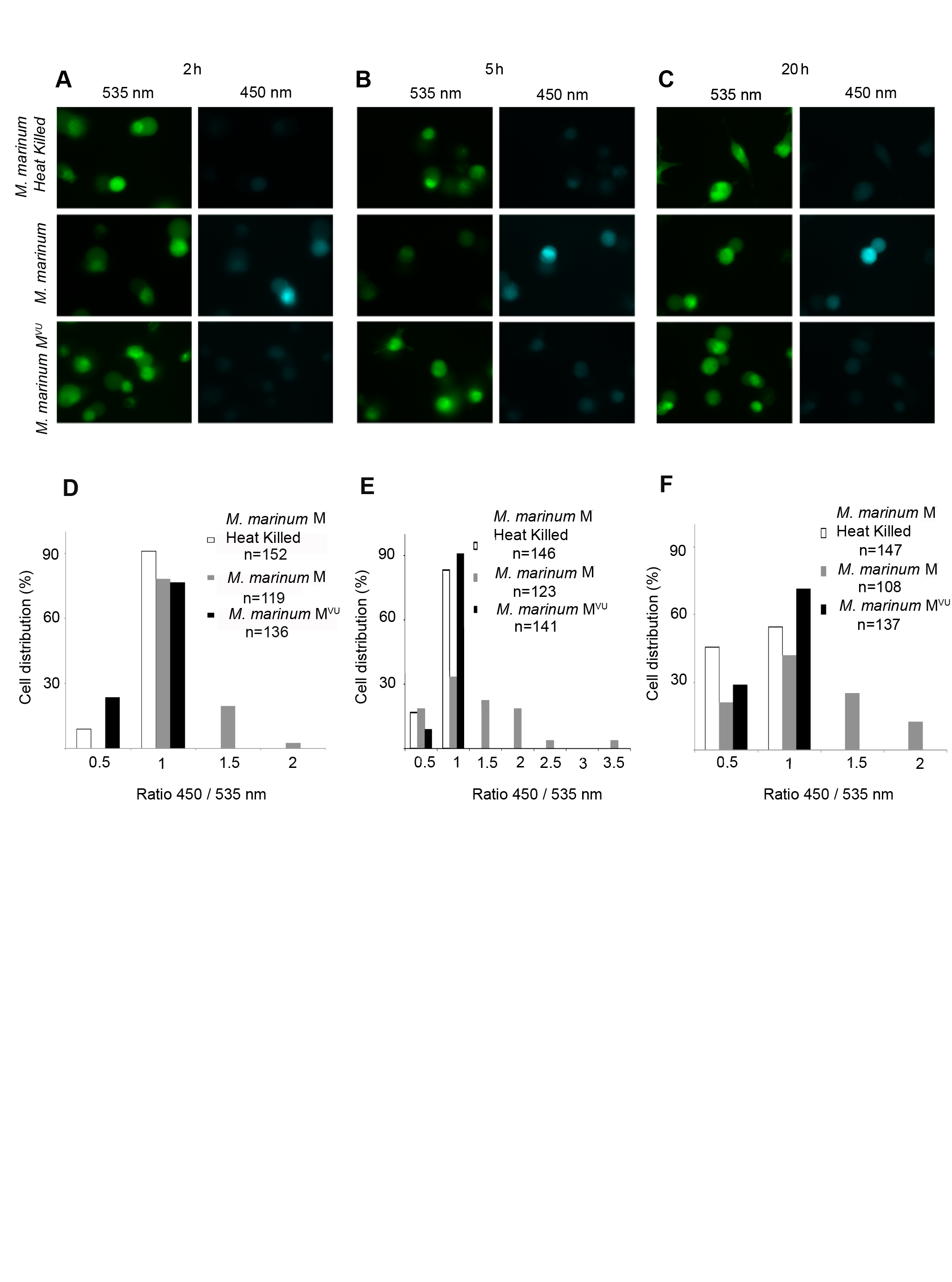

Supplement: Figure S3 — Cytosolic access of M. marinum depends on secretion of ESAT-6/CFP-10 effectors at early time points of infection. THP-1 cells were infected with M. marinum heat killed, M. marinum or M. marinum MVU at a MOI of 1 for 2 h (A,D), 5 h (B,E) or 20 h (C,F). Cells were then loaded with the CCF-4 molecule for 2 h. After PFA fixation, cells were imaged on a fluorescence widefield microscope (Nikon Ti) with a 40X objective (A,B,C). Picture acquisition was achieved randomly and automatically for each condition on 36 fields and further 450/535 nm intensity ratio measurements (D,E,F) were obtained through analysis by specialized algorithm on Metamorph software. Experiments were repeated 3 times with similar results. (TIF) [file ppat.1002507.s003.tif]

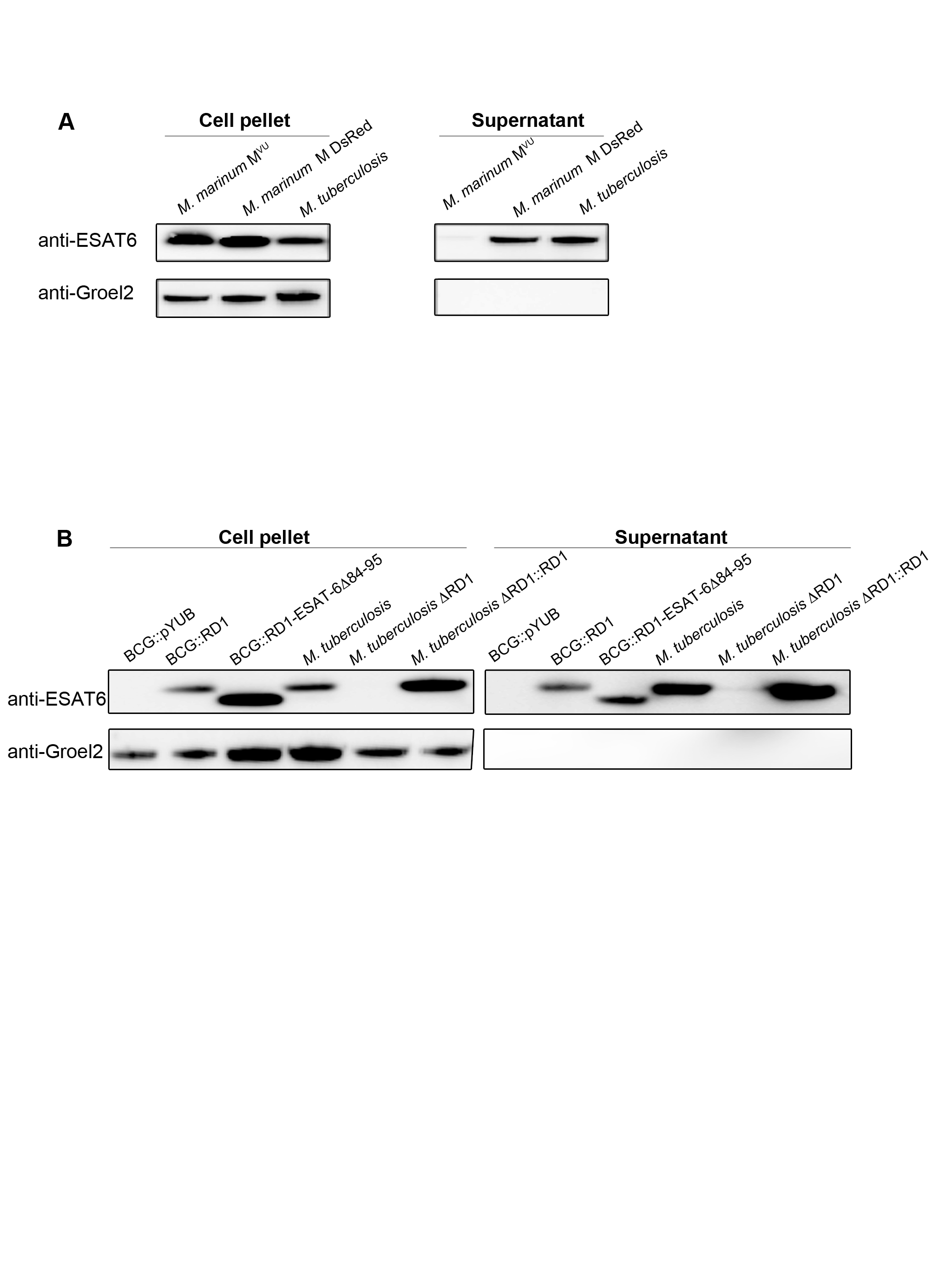

Supplement: Figure S4 — In vitro expression and secretion of ESAT-6 in different mycobacterial strains. Cell pellets and supernatants were subjected to SDS-PAGE and tested by Western blotting using a monoclonal anti-ESAT6 antibody or an anti-Groel2 antibody as lysis control. M. marinum MVU, M. marinum M DsRed; M. tuberculosis H37Rv (A), BCG::pYUB, BCG::RD1, BCG::RD1-ESAT-6Δ84-95, M. tuberculosis H37Rv, M. tuberculosisΔRD1, M. tuberculosisΔRD1::RD1 (B). (TIF) [file ppat.1002507.s004.tif]

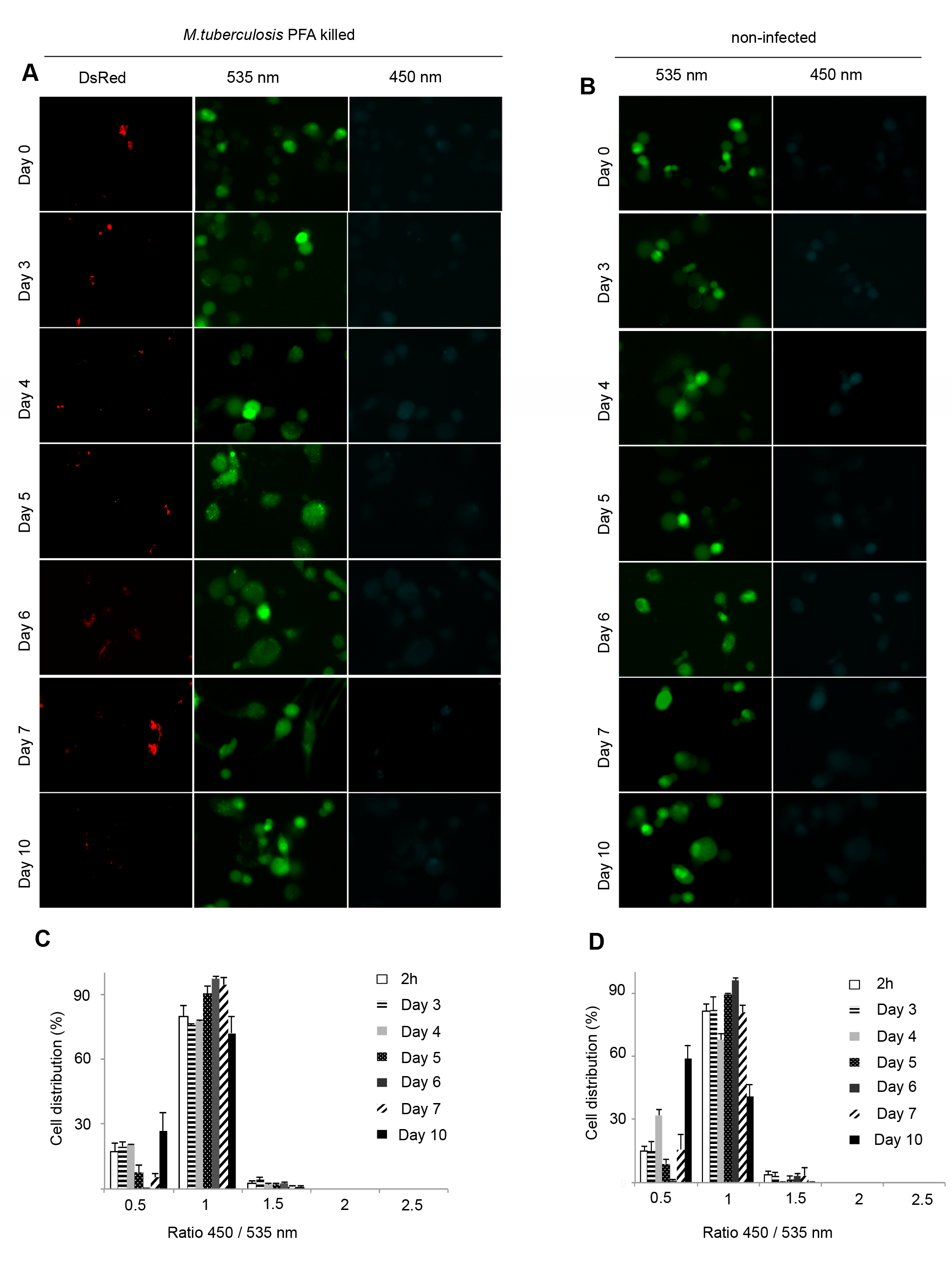

Supplement: Figure S5 — Time course of CCF-4 FRET signals in uninfected and PFA killed M. tuberculosis treated THP-1 cells. THP-1 cells were infected with M. tuberculosis DsRed PFA killed (A,C) or not infected (B,D) at a MOI of 1 for the indicated time and then loaded with the CCF-4 molecule for 2 h. After PFA fixation, cells were imaged on a fluorescence widefield microscope (Nikon Ti) with a 40X objective (A,B). Picture acquisition was achieved randomly and automatically for each condition on 49 fields and further 450/535 nm intensity ratio measurements (C,D) were obtained through analysis by specialized algorithms on Metamorph software. The plots were representative of 3 independent experiments. (TIF) [file ppat.1002507.s005.tif]

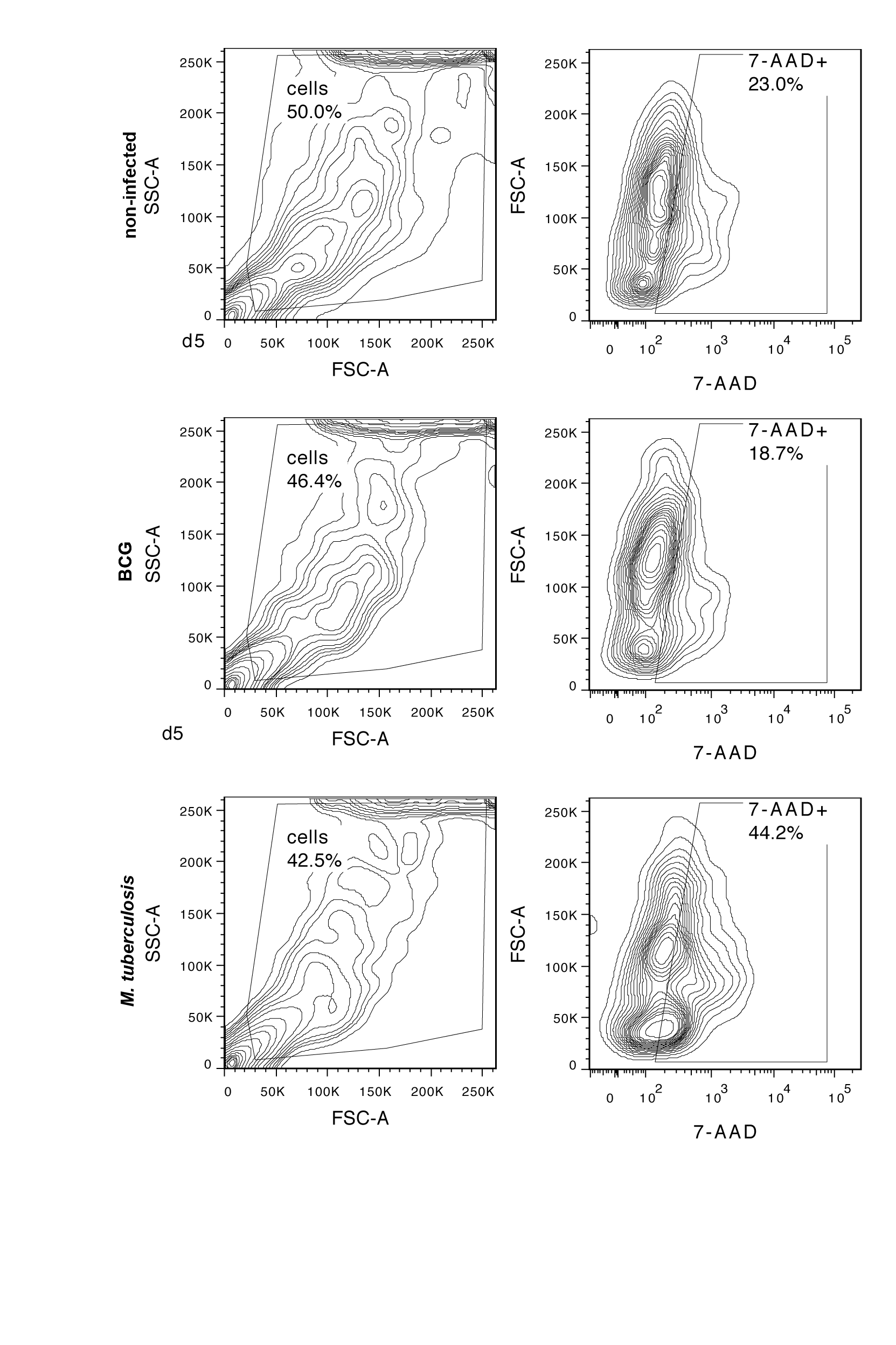

Supplement: Figure S6 — Evaluation of cell death in THP-1 cells by cytofluorometry. Cytofluorometry analysis of THP-1 cells infected with wild-type M. tuberculosis and BCG strains at day 5 post-infection. THP-1 cells were stained with 7-AAD (BD Pharmingen) (necrosis). (TIF) [file ppat.1002507.s006.tif]

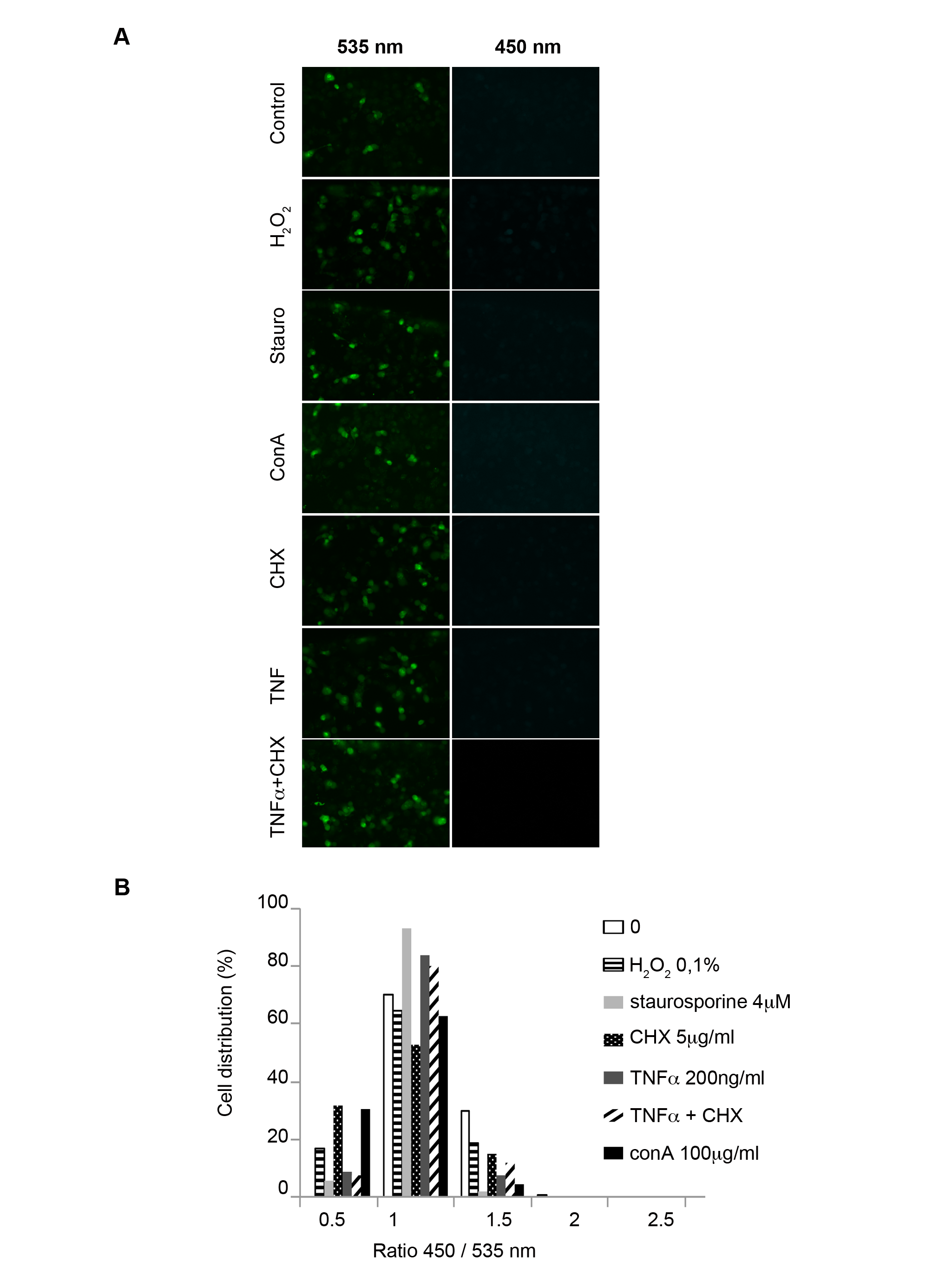

Supplement: Figure S7 — Apoptosis and necrosis do not influence the cleavage of the CCF-4 probe in THP-1 macrophages. THP-1 cells were subjected to different treatments for 18 h in order to induce either apoptosis or necrosis. Apoptosis was induced using 4 µM staurosporine, 5 µg/ml cycloheximide or 200 ng/ml TNF-α. Necrosis was induced using 0.1% H2O2, 100 µg/ml concanavalin A or cycloheximide/TNF-α combination. Cells were then loaded with the CCF-4 probe for 2 h. After PFA fixation, cells were imaged on a fluorescence widefield microscope (Nikon Ti) with a 40X objective (A). Picture acquisition was achieved randomly and automatically for each condition on 49 fields and further 450/535 nm intensity ratio measurements (B) were obtained through analysis by specialized algorithms on Metamorph software (TIF) [file ppat.1002507.s007.tif]

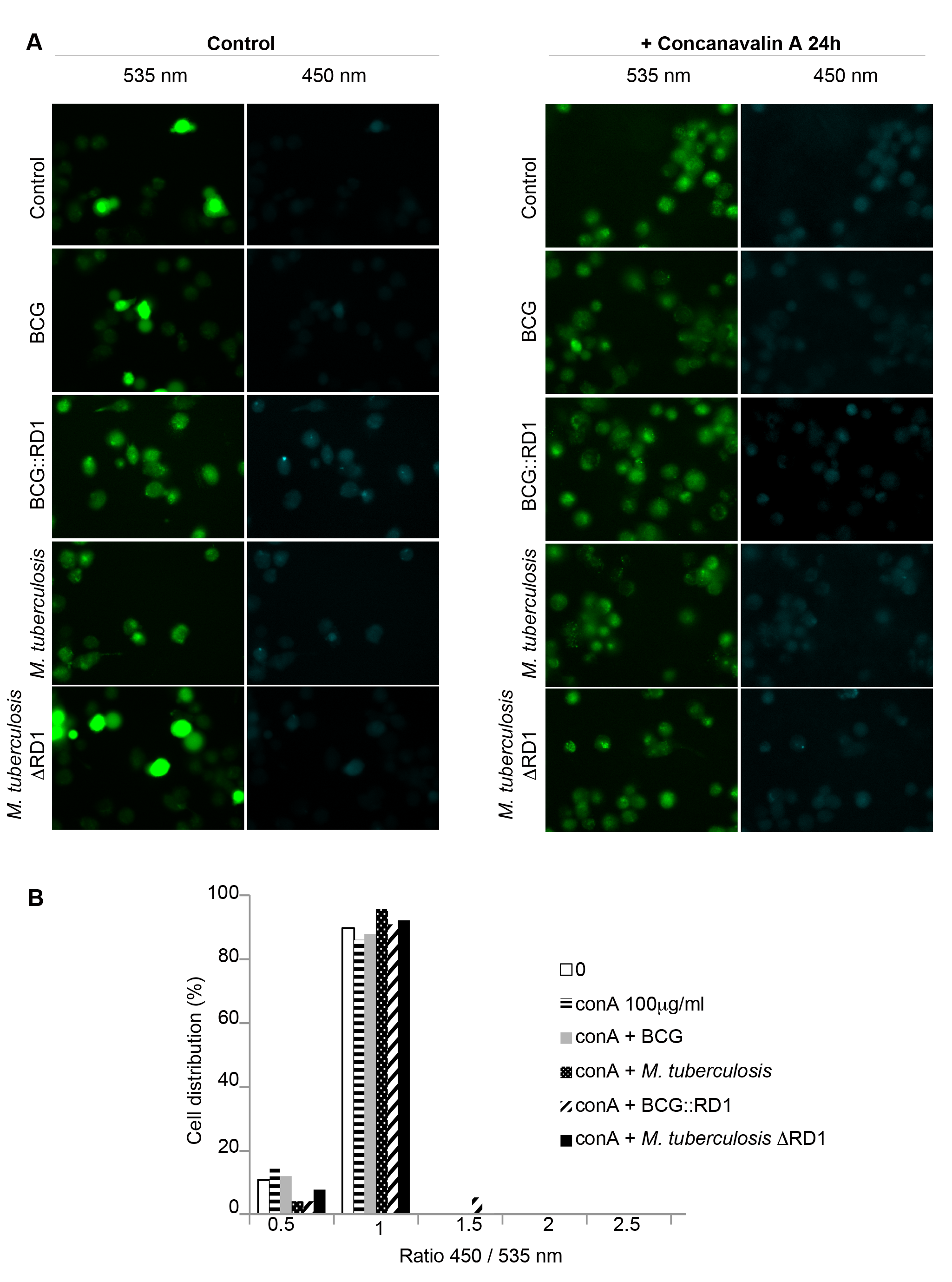

Supplement: Figure S8 — Necrosis induction does not influence the subcellular localization of mycobacteria. THP-1 cells were non infected (control) or infected with BCG, BCG::RD1, M. tuberculosis or M. tuberculosisΔRD1 at a MOI of 1 for 2 h. Then, necrosis was induced using 100 µg/ml concanavalin A for 24 h. Cells were then loaded with CCF-4 molecule for 2 h. After PFA fixation, cells are imaged by fluorescent widefield microscope Nikon Ti with 40X objective (A). Picture acquisition was achieved randomly for each condition on 49 fields and further 450/535 nm intensity ratio measurement (B) was obtained through analysis by a specialized algorithm on Metamorph software. (TIF) [file ppat.1002507.s008.tif]

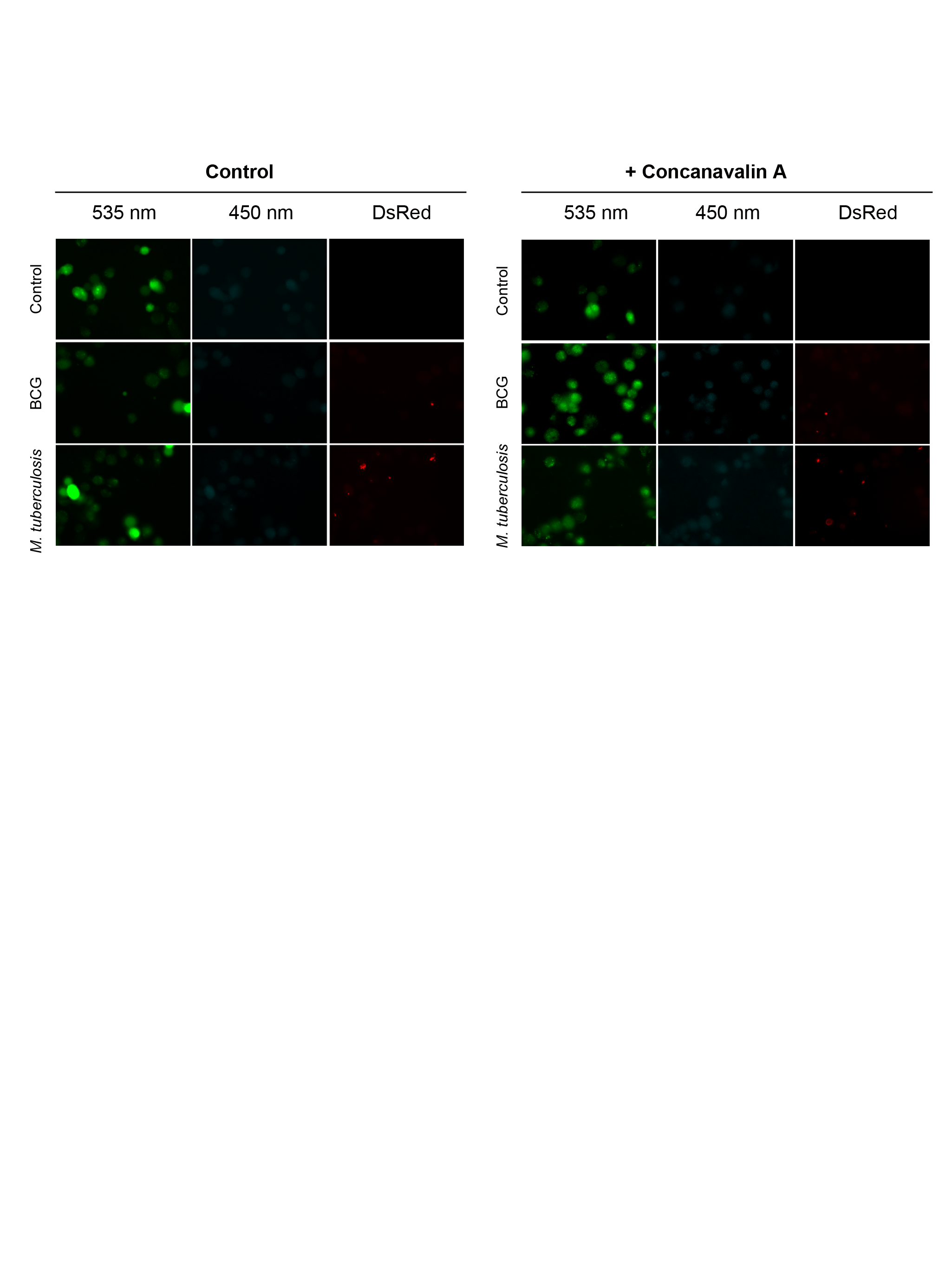

Supplement: Figure S9 — Necrosis induction in BCG or M. tuberculosis infected cells does not result in bacterial contact with host cytoplasm. THP-1 cells were non infected (control) or infected with BCG DsRed or M. tuberculosis DsRed at a MOI of 1 for 2 h. Then, necrosis was induced using 100 µg/ml concanavalin A for 24 h. Cells were then loaded with CCF-4 molecule for 2 h. After PFA fixation, cells were imaged by fluorescence widefield microscope Nikon Ti with 40X objective. (TIF) [file ppat.1002507.s009.tif]
